# Criminal Legal System Experiences Among Families Receiving Home Visiting Services: A Scoping Review of the Literature

**DOI:** 10.1007/s11121-025-01798-8

**Published:** 2025-03-24

**Authors:** Rebecca J. Shlafer, Zariyah Mohammed, Anam Hasan, Erin E. Reardon, Joshua P. Mersky, Laurel Davis, Allison L. West, Dylan B. Jackson

**Affiliations:** 1 University of Minnesota, Minneapolis, MN USA; 2 University of California Irvine, Irvine, CA USA; 3 Emory University, Atlanta, GA USA; 4 University of Wisconsin – Milwaukee, Milwaukee, WI USA; 5 Johns Hopkins Bloomberg School of Public Health, Baltimore, MD USA

**Keywords:** Early Childhood, Family Home Visiting, Criminal Legal System, Scoping Review

## Abstract

Each year, millions of families with children in the United States (US) come into contact with the criminal legal system (CLS), the deleterious consequences of which are well documented. Families exposed to the CLS often face many stressors and may benefit from supports and services designed to enhance parent–child relationships and connect them to health-promoting resources and services. Early childhood family home visiting (FHV) is a two-generation strategy to support pregnant women and families with infants and young children, many of whom encounter the CLS. Yet, little is known about the CLS experiences of families receiving FHV. This scoping review summarizes the published research on CLS experiences among FHV-enrolled families in the US. Seven online databases were used to identify research published between 1967 and 2022. Following PRISMA guidelines, articles were required to focus on FHV and CLS involvement. Twenty-eight articles met inclusion criteria; five were systematic reviews or meta-analyses, 22 were primary sources with quantitative measures of CLS, and one was a qualitative study. Among the primary quantitative sources, more than half (55%) included CLS measures to describe the sample and the others included CLS variables as outcomes. CLS involvement was a common experience among families receiving FHV services. This scoping review provides an important first step in describing the existing research on FHV participants’ CLS involvement and can inform future efforts to serve this group of families.

**Supplementary Information:**

The online version contains supplementary material available at 10.1007/s11121-025-01798-8.

## Introduction

In the United States (US), more than 6.75 million adults are currently under criminal legal system (CLS) surveillance, including confinement in prisons, jails, and detention centers, as well as under community supervision (e.g., probation, parole) (Carson, 2022; Kaeble, 2021; Zeng, 2021), with millions more reporting prior contact with the CLS. Data from the Bureau of Justice Statistics estimates that 48.1% of people incarcerated in state or federal prisons have at least one minor child, including 47% of male prisoners and 58% of female prisoners (Maruschak et al., 2021). In total, over five million children—5% of the US child population under the age of 6—have had a parent who lived with them go to jail or prison (Murphey & Cooper, 2015). In addition to the incarcerated parents with minor children, there are also nearly 58,000 admissions of pregnant people to jails and prisons each year (Sufrin et al., 2019, 2020).

Structural racism and systemic marginalization have contributed to markedly disproportionate rates of incarceration among adults who are Black, poor, living in rural areas, or have lower levels of educational attainment (Alexander, 2011; Muentner et al., 2022, 2023; Murphey & Cooper, 2015; Wildeman, 2009). As such, children of color experience both parental incarceration and intersecting aspects of disadvantage (e.g., housing instability, economic insecurity) at significantly higher rates than their white peers (Murphey & Cooper, 2015). In addition to structural disadvantages faced by families with CLS involvement, parental incarceration is a known adverse childhood experience (ACE) that often co-occurs with many other ACEs (e.g., parent substance abuse, parent mental health challenges) (Muentner et al., 2023; Turney, 2018), potentially compounding its risks for poor outcomes among children and families.

While there are many methodological challenges to estimating the impact of CLS contact on children and families, prevailing theory and evidence suggest that CLS contact is typically highly stressful, disruptive, and destabilizing, and undermines their health and well-being. A parent’s contact with the CLS can heighten parenting stress (Jackson et al., 2022) and interfere with secure attachment relationships between parents and children (Makariev & Shaver, 2010; Shlafer & Poehlmann, 2010), a foundational aspect to healthy child development (Benoit, 2004; Ranson & Urichuk, 2008). Compared to their peers, children of incarcerated parents experience greater psychological distress (Davis & Shlafer, 2017) and mental and behavioral health challenges (Jackson et al., 2021; Kjellstrand et al., 2018; Ruhland et al., 2020), more chronic health conditions and developmental disorders (Jackson et al., 2021), poor eating behaviors (Jackson & Vaughn, 2017), poor sleep health (Hiolski et al., 2019; Jackson & Vaughn, 2017), greater child health care burden and unmet healthcare needs (Jackson et al., 2022; Turney, 2017), and worse academic outcomes (Testa & Jackson, 2021; Turney & Haskins, 2014).

For the adults in a family, having another family member incarcerated is often highly stressful (Geller et al., 2009)—so much so that it has been described as “doing time together” (Comfort, 2008). A family member’s incarceration may lead to strain on relationships, financial difficulties, housing insecurity, among other stressors related to the unpredictability and complexity of the CLS. Even for formerly incarcerated individuals, the stress of returning to the community includes challenges reintegrating into civic society and reestablishing ties with friends and family, all while facing compounding formal and informal barriers to personal and family well-being and health (Brayne, 2014; Lee et al., 2014; Pager, 2003). For all of these reasons, households impacted by CLS involvement may benefit from supports and services that address these stressors and challenges, enhance parent–child connections, and promote health and well-being.

### Family Home Visiting (FHV): A Two-Generation Service Strategy

Early childhood family home visiting (FHV) is a voluntary, home-based, two-generation service strategy aimed at improving health outcomes for families living in communities most vulnerable to poor child and family outcomes. FHV staff provide social, emotional, health, and parenting information and support for families during pregnancy and through the first few years of a child’s life (Bilukha et al., 2005; Duffee et al., 2017; Minkovitz et al., 2016). During this sensitive period, home visitors are poised to address health and parenting needs, stressors, and challenges identified by the family to improve the home environment, promote parent–child attachment and healthy child development, prevent child abuse and neglect, and support other positive behaviors that are related to family health and well-being (Bilukha et al., 2005; Sar et al., 2010). Critically, home visiting programs achieve positive outcomes not only by supporting and educating families, but also through strategic and targeted referrals to other community-based resources and services (Duggan et al., 2018).

FHV is implemented widely; services are available in every U.S. state and territory, and in many tribal communities (National Home Visiting Resource Center, 2023). FHV services were expanded following the passage of Maternal, Infant, and Early Childhood Home Visiting (MIECHV) legislation under the 2010 Affordable Care Act, which invested $1.5 billion toward the implementation of evidence-based program models. The MIECHV program has been reauthorized on multiple occasions, and in 2022 Congress increased its scheduled funding levels to rise from $500 million in fiscal year (FY) 2023 to $800 million in FY 2027. In FY 2023, MIECHV served over 139,000 parents and children, 92% of whom had household incomes at or below 200 percent of the Federal Poverty guidelines (Maternal and Child Health Bureau, 2024a). Federal legislation mandates that states and territories spend most MIECHV funds to implement evidence-based models that have been evaluated based on stringent criteria (Adirim & Supplee, 2013). As of this writing, 24 models have met these criteria, including widely disseminated programs such as Healthy Families America (HFA), Parents as Teachers (PAT), and the Nurse Family Partnership (NFP).

Although MIECHV is the primary source of federal funding for FHV, states, territories, and local communities often leverage funding from additional sources such as state or local general revenue, private foundation funds, TANF, Medicaid, and Title IV-E funds under the Family First Prevention Services Act. In some cases, programs braid MIECHV dollars with these funding sources, whereas other programs operate without MIECHV funding. As a result, the total capacity for evidence-based FHV services in the US is substantial. To illustrate, in 2022, more than 270,000 families received evidence-based FHV services nationwide (National Home Visiting Resource Center, 2023). There are also many FHV programs in the US that implement models that are not recognized by MIECHV as an evidence-based model. Many of these programs are locally developed and designed to meet the needs of a particular community or families in special circumstances. The next section considers the potential for FHV programs to meet the special needs of CLS-involved families.

### The Potential of FHV to Serve Families Impacted by the CLS

FHV programs commonly serve families experiencing some of the same challenges and hardships as families impacted by the CLS—including poverty, low educational attainment, and substance use—yet little is known about the degree to which these programs serve this population. FHV is likely well-positioned to support families with past or current CLS involvement in a number of ways. Not unlike other families who receive FHV services, families with CLS involvement may benefit from direct services to promote healthy parent–child interactions and attachment (Makariev & Shaver, 2010; Shlafer & Poehlmann, 2010), as well as referral and coordination with needed community resources, such as housing (Geller & Franklin, 2014), transportation (Turney, 2019), health care (Jackson et al., 2022), and mental health support (Davis & Shlafer, 2017). Yet, families impacted by the CLS are likely to experience acute barriers to accessing and navigating these resources. For example, depending on the jurisdiction and criminal conviction, impacted families can experience collateral consequences that limit or prohibit access to certain types of employment (e.g., restrictions on working in certain settings), housing (e.g., legal barriers to where people can live), and economic benefits (e.g., bans from public assistance and food stamps). Home visitors may play an especially vital role in helping CLS-involved families with accessing resources that meet their complex needs and navigating the CLS and other intersecting service systems (e.g., arrange transportation to court hearings, schedule court-ordered assessments).

Beyond supporting families with current or past CLS involvement, FHV is a two-generation strategy that has potential to reduce the risk of future CLS involvement for parents and their children. There is ample evidence that other early childhood interventions (e.g., center-based preschool programs, evidence-based treatments for emotional and behavioral disorders) can reduce children’s risk of delinquency and becoming involved in the CLS themselves later in life (Cannon et al., 2018; Reynolds et al., 2004; Schweinhart, 2013; Welsh & Farrington, 2015), but little is known about whether and how often FHV programs have similar impacts. Conceptually at least, the MIECHV Program could act as a catalyst for generating knowledge related to the impact of FHV programs on CLS involvement. By law, MIECHV grantees must demonstrate improvements in at least four of six benchmark areas, one of which, “reduction in crime or domestic violence.” However, MIECHV-funded programs are only required to collect data on one performance measure in this domain, which indicates the percentage of primary caregivers who are screened for intimate partner violence within 6 months of enrollment (Maternal and Child Health Bureau, 2024b). In other words, although crime reduction is a stated goal of the MIECHV Program, grantees that receive MIECHV funding are not required to assess whether the families they serve are involved in the CLS, much less whether they have an impact on CLS involvement.

### The Current Study

Despite the potential for FHV to serve and improve outcomes for families impacted by the CLS, the extent to which this has been studied is unknown. A scoping review is therefore the ideal tool to determine the scope and coverage of the existing body of literature and to examine and summarize how research has been conducted on the topic to date (Munn et al., 2018). To that end, the purpose of this scoping review is to summarize the published research examining the CLS experiences of families participating in FHV programs in the US, to identify key characteristics of this literature, and to identify gaps in existing knowledge to inform future research in this area.

## Methods

### Protocol and Registration

The scoping review was based on the Preferred Reporting Items for Systematic Reviews and Meta-analysis Protocols (PRISMA) Statement extensions for systematic review protocols and scoping reviews (PRIMSA-ScR), and materials developed by The Campbell Collaboration. The protocol was developed a priori and registered (Reardon, 2022) at the Open Science Framework (Center for Open Science, 2024).

### Eligibility Criteria

Articles that were included in the review were required to have a measure or outcome focused on both FHV programs and CLS involvement (any history of arrest, conviction, incarceration, probation, parole, or juvenile justice) among participants, including caregivers (before, during, or after participation in FHV services) or their children in adolescence or adulthood (after participation in FHV services). Publications were included if they were published between 1967 and 2022, set in the US, involved at least one of the aforementioned CLS measures, and evaluated expectant families (both caregivers and children) or families with children under the age of 6 participating in a voluntary FHV program. Case studies, systematic reviews, qualitative and quantitative studies, dissertations, and conference abstracts were included in the review. Because home visiting programs that prioritize families with current or past involvement in the CLS may be newer and less well-established, we included studies of both non-evidence-based FHV programs and evidence-based programs. Furthermore, while many evidence-based FHV programs are intended to be primary prevention programs (engaging with families before caregivers engage in high-risk behavior such as abuse or neglect) (Home Visiting Evidence of Effectiveness (HomVEE), 2024), this review was not limited to these programs, as it also included FHV services that had targeted high-risk populations for secondary prevention (e.g., targeting caregivers with substance use disorders).

Papers were excluded from the review if they failed to meet the predetermined criteria. Specifically, publications were not considered if they focused on home visiting programs that targeted a single generation (i.e., only parents or children) or served children age 6 or older. Further, because the CLS in the US differs from justice systems in other countries in many ways, we excluded studies that were implemented outside of the US. Papers were also excluded if the service providers were the focus of analysis, if the only CLS measure was intimate partner violence, or if they did not include or review original research (e.g., an editorial, commentary/position paper, court case).

### Information Sources

The search strategy to identify relevant articles was built by a health sciences librarian after consultation with the study team and tested for sensitivity in Ovid MEDLINE using medical subject headings (MeSH), keywords, and synonyms, then translated to an additional seven databases: Embase and PsycINFO via OVID; Social Services Abstracts; Cochrane Library via Wiley; CINAHL via EBSCOhost, Sociological Abstracts, and Criminal Justice Database. Searches were run from the inception of each database through March 15, 2022. The full search strategy can be found in the Appendix.

### Selection of Sources of Evidence

Two independent reviewers were responsible for each stage of the selection process (i.e., abstract/title review, full-text review, extraction). After removing duplicates, 493 unique titles and abstracts were screened in the Covidence software (Covidence, 2023) using the predetermined inclusion criteria. Of these 493 abstracts, 404 were excluded because they did not meet inclusion criteria (e.g., were not focused on FHV, did not include a CLS measure), resulting in 89 that met inclusion criteria and advanced to full-text review. During the full-text review phase, two independent reviewers closely analyzed the text of each study to determine whether its measures met the scoping review’s objectives. If there were conflicting decisions about whether an article was to be excluded or included, then a third independent reviewer helped resolve conflicts to arrive at consensus.

### Data Extraction and Data Items

Before data extraction, we separated review articles from primary research studies (i.e., those that analyzed new or existing data). Data were extracted from the primary research studies in Covidence. The data extraction form was developed based on the required reporting elements for scoping reviews and was tested on five articles, then revised for consistency among the extractors before being used to extract the remaining articles. The extracted data included the following elements: study title, authors, location, aims, design (randomized control trial, cohort, cross-sectional, case control, systematic, qualitative, prevalence, case series, case report, economic evaluation, quasi-experimental, or other), a brief description of participants, number of participants, year the study was conducted, and whether participants received home visiting services from an evidenced-based model, as defined by the MIECHV statute. In addition, the specific program name (e.g., Attachment and Biobehavioral Catch-Up, HFA, NFP) was recorded for MIECHV model programs or “other” for non-model programs. CLS measures were extracted and categorized as either independent (e.g., demographic characteristic) or dependent variables and coded from a list of 10 terms developed by the study team (police stops, arrest, conviction, jail, prison, incarceration, probation, parole, delinquency, CLS involvement, other). In addition, information was extracted from each study to ascertain which participants were involved in the CLS (e.g., mothers, youth), when CLS involvement was assessed (e.g., pre- or post-intervention), how CLS involvement was measured (e.g., self-report, official records), and major study findings.

### Synthesis of Results

The studies were then synthesized within four groups: (1) review studies, (2) qualitative studies, (3) quantitative studies in which CLS was a demographic characteristic or independent variable, and (4) quantitative studies in which CLS was a dependent variable. Within each of these four groups, we sought to identify key characteristics of the literature and existing gaps.

## Results

**Fig. 1:**
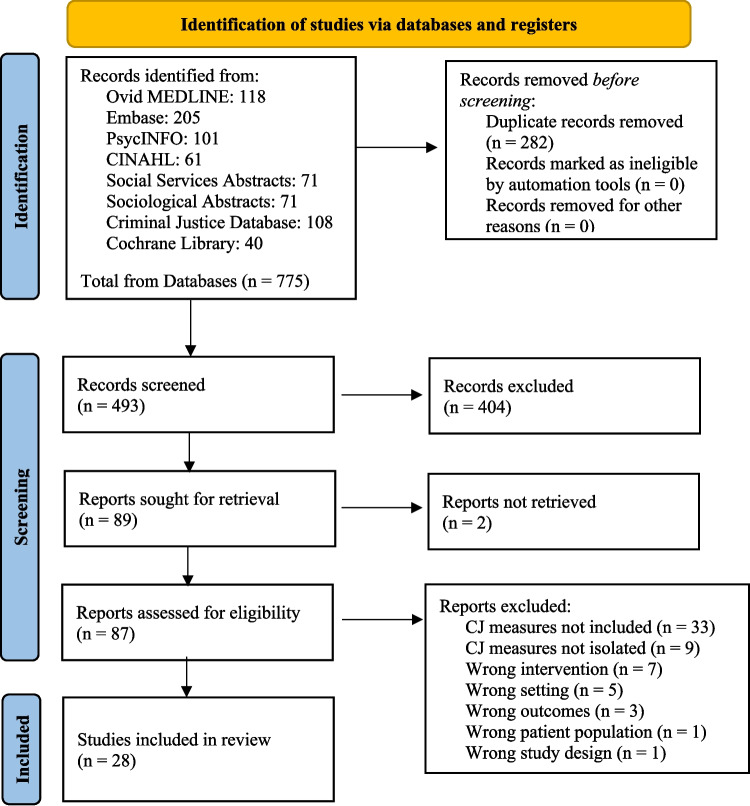
PRISMA flow diagram

In total, 28 articles met the inclusion criteria and addressed the research aim (see Fig. 1). The included studies were published between 1994 and 2022. Of the 28 articles, five reviewed existing evidence and 23 were primary studies.

Among the five non-primary studies (Table 1), two were systematic reviews (Bernazzani et al., 2001; Cohen et al., 2010); two were other types of reviews which summarized evidence from NFP, specifically (Miller, 2015; Olds, 2007), and one was a meta-analysis (Farrington & Welsh, 2003). Overall, these reviews demonstrated a positive impact on CLS outcomes for mothers and youth, though they often cite the same few randomized control trials of NFP by Olds and colleagues (Kitzman et al., 2019; Olds et al., 1997, 1998). In one exception, Cohen and colleagues’ (2010) review of early childhood interventions cited evidence from a trial of the Infant Health and Development Program demonstrating a positive impact on juvenile arrest (McCormick et al., 2006).

**Table 1:** Systematic reviews and meta-analyses that met inclusion criteria

Authors (year)	Study aims and key findings
Bernazzani et al. (2001)	This review summarized the findings from seven randomized or quasi-experimental trials assessing the impact of early parenting and FHV on behavior problems and delinquency in children. The review was limited to studies that engaged families with young children (under the age of 3) when the intervention began. Six of the seven studies included in the review examined home visiting programs. The authors originally sought to assess the impact of programs on children’s subsequent delinquent behavior. However, the scope of the study was broadened to include other measures of children’s disruptive behaviors (e.g., self-reported delinquency, parent-reported disruptive behavior) because the original search only revealed one study that assessed children’s delinquency. The only study included in the review that examined criminal justice measures was from Olds et al. (1986, 1998), which included measures of children’s experiences of police stops, arrest, convictions, and delinquent acts at age 15
Cohen et al. (2010)	This review of the literature summarizes early childhood interventions (including home visiting) that address a number of adverse outcomes, including crime and delinquency, drug and alcohol abuse, child abuse and neglect, and poor health outcomes. In their summary of home visiting programs, the authors summarize the existing literature on the impact of FHV on reducing crime and violence (e.g., child delinquency, antisocial behavior, maternal arrest). Their review of FHV almost exclusively relies on evidence from trials of Nurse Family Partnership, including evidence that participating mothers had fewer arrests (Olds et al., 1997) and youth had fewer arrests, convictions, and violations of probation. The authors also note findings from a trial of the Infant Health and Development Program, which demonstrated that program participation reduced the likelihood of juvenile arrest (McCormick et al., 2006). In addition to their review of early childhood interventions, the authors include cost calculations for the value of lifetime costs imposed on society for each of the “social ills” (p. 391) they examined (e.g., crime, child abuse and neglect, poor health). The authors find that lifetime criminal offending poses the largest costs to society. They conclude that well-designed early childhood interventions have the potential to have significant “monetary value of saving a high-risk youth from a lifetime of adverse outcomes such as crime and delinquency” (p. 423)
Farrington and Welsh (2003)	This paper presents a review and meta-analysis of the effectiveness of family-based crime prevention programs. Forty studies met inclusion criteria: (a) the family was the focus of the intervention, (b) there was an outcome measure of delinquency or antisocial child behavior, (c) the evaluation used a randomized or well controlled experiment and (d) the original sample size was at least 50. Of the 40 studies included in the review, only four home visiting studies met criteria for inclusion (Cullen, 1976; Stone, Bendell & Field, 1988; Kitzman et al., 1997; Olds et al., 1998). Olds et al., 1998 is the only study reviewed that found significant effects of self-reported arrests of children whose mothers received home visits
Miller (2015)	This article systematically reviewed 39 evaluation reports from the Nurse Family Partnership (NFP) to project the long-term program impacts on 21 outcomes, including youth crime and arrest. Miller summarizes data from the Elmira trial of NFP which found that NFP reduces youth arrest by 44.6% at ages 11 through 19. Using these data, Miller concludes that by 2031, NFP enrollment between 1996 and 2013 will prevent 90,000 violent crimes and nearly 600,000 property and public order crimes by youth, as well as 36,000 youth arrests
Olds (2007)	This paper summarizes key findings from 27 years of data from the Nurse-Family Partnership (NFP). In terms of criminal justice measures, Olds summarizes findings from a prior empirical study (Olds et al., 1997) demonstrating positive impacts on maternal arrest and youth outcomes (i.e., fewer arrests, convictions, and violations of probation) at the 15-year follow-up

Among the 23 primary studies, only one was a qualitative study (Fauth & Winestone, 2021). In their study, Fauth and Winestone conducted in-depth interviews with parents, home visitors, and CLS professionals to examine implementation features and practices among two distinct home visiting programs that specifically served young parents with CLS involvement. Fauth and Winestone found that home visitors’ strength-based, trauma-informed approach, combined with their knowledge of the CLS, was particularly valuable in supporting parents and helping them navigate the complexities of the CLS.

We extracted key information from each of the remaining 22 primary quantitative studies. More than half (*n* = 13, 59%) of the studies were randomized controlled trials; the remainder were cohort studies (*n* = 6), other designs (*n* = 2), or case control designs (*n* = 1). Half (*n* = 11) of the studies included participants from evidence-based models that were eligible for MIECHV funding, seven of which were drawn from NFP, two from HFA, and two from PAT. The remaining 11 studies included participants from programs that were not eligible for MIECHV funding. The sample sizes ranged from 37 to 6792.

Next, we summarized the primary quantitative studies featuring CLS variables as demographic characteristics (*n* = 12, 55%; Table 2) or outcome measures (*n* = 10, 45%; Table 3). There were 12 studies measuring CLS involvement as a demographic characteristic (Table 2). These 12 studies were published between 1994 (Black et al., 1994) and 2022 (Traube et al., 2022). These studies largely relied exclusively on parent (most often maternal) reports of CLS. There were, however, two exceptions to this pattern—one study reviewed child protection records (Duffy et al., 2015) and another supplemented maternal reports with home visitor case notes (Thomas et al., 2018). Most of these studies measured only mothers’ CLS involvement. The studies varied in what they assessed related to CLS involvement, though nearly all (*n* = 10) assessed incarceration history. Notably, however, most studies did not specify whether the incarceration was in jail or prison, or consider other aspects of the incarceration experience (e.g., duration, frequency, timing in the life course). More than half (*n* = 7) of these studies included CLS measures at only one study timepoint; the other five included measures at two or more study timepoints.

**Table 2:** Primary quantitative studies that met inclusion criteria and included criminal legal system (CLS) measures as demographic characteristics

Authors (year)	Study aims/goals	Study design^a^	*N*	FHV program^b^	CLS measures	Major findings related to CLS measures
Black et al. (1994)	Test the efficacy of a home-based intervention program in promoting positive behaviors and attitudes among women who use drugs and development among their children	RCT	60	Other	Maternal self-report of incarceration at baseline and 18 months	At baseline, 55% in intervention group and 69% in the comparison group had an incarceration history. At study completion, 55% of intervention had an incarceration experience compared to 65% of comparison. Among those lost to follow-up, 55% in the intervention and 83% in the comparison had an incarceration experience
Black et al. (2006)	Examine (1) to examine whether the mentoring intervention was effective in preventing second births within 2 years of the adolescent mother’s first delivery; (2) whether greater intervention participation increased the likelihood of preventing a second birth; (3) whether second births were better predicted from a risk practice perspective or a family formation perspective, based on information collected at delivery; and (4) how risk practices or family formation over the first 2 years of parent-hood were related to a second birth	RCT	149	Other	Maternal self-report of arrest and jail incarceration at baseline and 24-month follow-up	At baseline, 17% of mothers had been arrested; mothers who had a second baby were more likely to have been arrested at baseline. At 24 months, 6.7% of the sample had been arrested. Arrest rates decreased from 17% at baseline to 6.7% at 24 months. At baseline, rates of “jailed” were low (11% among the second baby group; 3% among the no second baby group). At 24 months 6.7% of the sample had been jailed (4% among the second baby group; 2.5% among the no second baby group)
Cullen et al. (2010)	Examine the effects of a credentialed home visitation program on the parenting attitudes and practices of a sample of at-risk parents; examine the social and emotional competence of children whose parents successfully completed the program	Cohort	64	HFA	Participant self-report of family criminal history at baseline	64% reported criminal history at baseline
Demeusy et al. (2021)	Study 1 aims to examine maternal depression as a mechanism in the effects of home visiting on parenting and offspring outcomes at post-intervention. Study 2 examines the sustained effects of home visiting (3–7 years following post-intervention) on negative and positive parenting practices, as well as child symptomatology and self-regulation, when the child is school age	RCT	Study 1: 232 Study 2: 87	Other	Maternal self-report of incarceration at follow-up	In Study 2, 35% of mothers reported someone in their family or someone close to them had experienced incarceration
Duffy et al. (2015)	Examine the relationship between parental risk factors and the substantiation status and number of child protective services reports in families in a statewide prevention program	Case Control	1125	PAT	Review of child protective services records of maternal and paternal criminal history during intervention period	19% of mothers and 59% of fathers had a criminal history. Adjusted models revealed that families with a substantiated versus unsubstantiated first report with child protective services were more likely to have a maternal criminal history. Paternal criminal history was not associated with risk for substantiated vs. unsubstantiated in either unadjusted or adjusted models
Eddy et al. (2020)	Examine whether the program as practiced (i.e., the Full Program condition) leads to positive short-term outcomes for families compared to respite care and referral alone	RCT	180 dyads	Other	Primary caregiver self-report of arrest, and jail and prison incarceration before intervention	20% of primary caregivers had spent time in jail or prison
Mersky and Janczewski (2018)	Assess the racial/ethnic distribution of adverse childhood experiences (ACEs) and five other major adversities (food insecurity, homelessness, prolonged parental absence, peer victimization, and violent crime victimization) in a diverse sample of low-income women	Cohort	1523	Other	Maternal self-report at baseline of childhood history of household member incarceration	37.9% of the sample reported a childhood history of household incarceration; compared to white mothers, Hispanic mothers were less likely and American Indian mothers were more likely to report childhood history of household incarceration
Mersky et al. (2018)	Examine the prevalence and interrelations of 10 adverse adult experiences, including household events such as intimate partner violence and extra familial events such as crime victimization. Test the relation between cumulative adult adversity and later mental health problems, and examine whether adult adversity mediates the link between childhood adversity and mental health	Cohort	501	Other	Maternal self-report of jail incarceration of self, father or significant other at baseline	48.2% of the sample reported incarceration of a partner or spouse. At the bivariate level, partner incarceration/jail was positively correlated with all other measures of adversity, and maternal reports of depression, anxiety, and PTSD
Nair et al. (2003)	Assess the relationship between cumulative environmental risks and early intervention, parenting attitudes, potential for child abuse and child development in substance abusing mothers	RCT	161	Other	Maternal self-report of incarceration every 6 months until 24 months	20.5% of mothers endorsed being incarcerated at any point since the start of the study
Sampson et al. (2017)	Using secondary data from Healthy Families America, this study sought to provide in-depth description of predictors of elevated postpartum depression symptoms among participating mothers	Cohort	4979	HFA	Maternal self-report of criminal history at intake	22% of mothers reported a criminal history; criminal history was not related to risk for postpartum depression or any of the home environment measures
Thomas et al. (2018)	Describe conditions and dynamics in the lives of high-risk, low-income, Southern United States prenatal-interconceptional women in a home visiting program that promoted maternal health literacy progression	Cohort	37	Other	Maternal report and home visitor case notes regarding paternal incarceration at any point during the intervention	19% of mothers reported incarceration of the fathers of the babies
Traube et al. (2022)	Explore differences in early childhood home visitation service provision (enrollment and depression screening) among mothers receiving home visitation services prior to and after the onset of the COVID-19 pandemic	Cohort	6792	PAT	Maternal report at baseline of history of parental incarceration during the child’s lifetime	5% reported a history of parental incarceration during the child’s lifetime; no differences between groups (pre-pandemic vs. during pandemic). After the onset of the pandemic, mothers who reported that the family had a history of incarceration were more than twice as likely to meet criteria for depression (25%) than mothers who did not (9.8%)

Notes: ^a^*RCT*, randomized controlled trial; ^b^*HFA*, Healthy Families America; *PAT*, parents as teachers

**Table 3:** Primary Studies that Met Inclusion Criteria and Included Criminal Legal System (CLS) Measures as Outcomes

Authors (year)	Study Aims/Goals	Study Design^a^	*N*	FHV Program^b^	CLS Measures	Major Findings Related to CLS Measures
Eckenrode et al. (2001)	Investigate the relationship between child maltreatment and the early onset of problem behaviors among offspring in the Elmira Nurse Home Visitation Program	RCT	400	NFP	Youth self-report; parent report; official records at 15-year follow-up; youth arrest, conviction, and delinquency	17.9% of children experienced an early age onset of arrest. By their 15th year, fewer than 15% of this study's youth had a person in need of services (PINS) report filed, had been arrested, or had been convicted of a crime. Child maltreatment moderated the association between intervention and early onset outcomes. For youth in the comparison group, child maltreatment was associated with increases in the number of early onset problem behaviors. For the youth in the intervention group, there was no relationship between maltreatment and early onset problem behaviors."
Eckenrode et al. (2010)	Examine the effect of prenatal and infancy nurse home visitation on the life course development of 19-year-old youths whose mothers participated in the program	RCT	400	NFP	Youth self-report of youth arrest, conviction, and delinquency at 19-year follow-up	Among the 19-year-old youths, those visited by nurses during pregnancy and infancy were less likely to have ever been arrested or convicted than were those in the comparison group. Youths in the nurse-visited group also had fewer lifetime arrests and convictions than did those in the comparison group. Examination of treatment differences separately for boys and girls suggests that these effects were limited to girls. Specifically, nurse-visited girls were less likely to be arrested (10% vs 30%) and convicted (4% vs 20%) than were girls in the comparison group. There were no differences between nurse-visited boys and comparison boys in the lifetime likelihood of being arrested (46% vs 47%) or convicted (35% vs 39%). Intervention effect on number of arrests and convictions also varied by sex. Nurse-visited girls had fewer mean lifetime arrests (0.10 vs 0.54) and convictions (0.04 vs 0.37) than did girls in the comparison group; nurse-visited and comparison boys had similar rates of mean lifetime arrests (1.39 vs 1.37) and convictions (0.97 vs 0.91). Examination of age at first arrest in the proportional hazards model indicated that nurse-visited youths had a smaller risk of first arrest than did youths in the comparison group; this effect varied by sex. Nurse-visited girls had a smaller risk of first arrest than did comparison girls. Nurse-visited and comparison boys were similar in their risk of first arrest
Grant et al. (2003)	Examine the post-program follow-up status of mothers who abused alcohol and drugs heavily and received paraprofessional home visitation and advocacy for 3 years after delivery	Other	65	Other	Maternal self-report of jail and prison incarceration at end of intervention and 3-year follow-up	Between program exit and post-program follow-up there was a decrease in mothers jailed during the interval (67% during program versus 39% during follow-up)
Honig (2004)	Assess the longitudinal outcomes of the Family Development Research Program	Other	119	Other	Youth self-report; parent report; teacher report; youth probation, delinquency, and other recidivism at 10-year follow-up	The strongest findings of the 10-year follow-up were for juvenile delinquency; differences were found between program and control youth. Only 6% of the program youth in the follow-up sample as compared with 22% of the control youth were processed as probation cases. In addition, the severity of the offenses was much graver for the control youth
Kitzman et al. (2019)	Examine whether program would improve 18-year-old first-born youths’ cognition, academic achievement, and behavior and whether effects on cognitive-related outcomes would be greater for youth born to mothers with limited psychological resources and on arrests and convictions among females	RCT	742	NFP	Youth self-report; parent report of youth arrest and conviction at 18-year follow-up	There were no overall intervention vs. control differences in counts of arrests among youth. Nurse-visited females, as a trend, had fewer convictions than the control group
Olds et al. (1997)	Examine the long-term effects of a program of prenatal and early childhood home visitation by nurses on women's life course and child abuse and neglect. Determine the extent to which the beneficial effects of the program instituted early in the life cycle altered the life-course trajectories of the mothers through the child's 15th birthday	RCT	324	NFP	Maternal self-report of arrest and conviction at 15-year follow-up	Nurse-visited women had fewer arrests by self-report and arrest disclosed by New York State (NYS) records. Data from NYS showed that nurse-visited, low-SES, unmarried women had fewer actual arrests and fewer convictions
Olds et al. (1998)	Examine the long-term effects of a program of prenatal and early childhood home visitation by nurses on children’s antisocial behavior	RCT	315	NFP	Youth self-report; parent report; official records at 15-year follow-up; youth police stops, arrest, conviction, probation, and youth sent to corrections	Adolescents born to nurse-visited women (treatment group) reported more frequent stops by police, but fewer arrests and convictions and violations of probation; the arrest and convictions and probation violation effects were concentrated among children born to women who were unmarried and from low-SES families. For the subsample of children who lived in Chemung County for their entire lives, nurse-visited children had fewer official PINS records. Nurse-visited children whose mothers were unmarried and from low-SES families were reported by their parents to have been arrested less frequently than their peers in the comparison group. The effect of the program on arrests was not limited to any specific type of crime, although property crimes were more frequent and, therefore, accounted for a larger portion of the program effect on arrests overall
Olds et al. (2007)	Test the effect of prenatal and infancy home visits by nurses on mothers’ fertility and children’s functioning 7 years after the program ended at child age 2	RCT	743	NFP	Maternal self-report of arrest and jail incarceration at 9-year follow-up	Between 6–9 years, 2.5% reported being jailed vs. 3.7% in the nurse-visited group. There were no program effects on women’s arrests or being jailed
Petitclerc and Brooks-Gunn (2022)	Estimate the effects of two common early childhood intervention ingredients—home visits and center-based education—on juvenile justice involvement	RCT	1090	Other	Youth and caregiver report of youth police stops, arrest, and incarceration by 18-year follow-up	By age 18, 61.8% of youth in the sample (79% of boys, 46.4% of girls) reported every being stopped, questioned, or in trouble with the police. 12.3% of the sample (19.2% of the boys, 6.2% of the girls) ever went to jail, prison, or a juvenile correctional facility. 20.1% of the sample (31.4% of boys, 10% of girls) were ever arrested by the police. Results showed an intent-to-treat effect on boys’ risk of being arrested. Specifically, boys’ odds of being arrested were reduced by 57% if they were assigned to the intervention group vs. the control group. No effects were found on girls’ justice involvement. Analyses of dosage effects showed that, for both sexes combined, participation in the center-based educational component decreased the odds of being stopped by the police by 3% for each month of services. For boys only, the odds of being arrested decreased by 4% with each month of home visits and by 4% with each month of center-based educational services
Shlafer et al. (2012)	Examine the relative effects of maternal jail time, conviction, and arrest and earlier risks on adolescent outcomes at age 15	RCT	330	NFP	Maternal and child self-report at 15-year follow-up; maternal police stops, arrest, conviction, and jail incarceration; youth police stops, arrest, conviction, incarceration and custody	Controlling for treatment status, prenatal risks, and child gender, adolescents whose mothers had a history of arrest were more likely to have been stopped by police or been sent to corrections. Compared to mothers with no criminal history, adolescents whose mothers had been convicted were more likely to have been stopped by the police, been sent to youth corrections, or been identified PINS. Adolescents were more likely to have been stopped by police, been sent to youth corrections, if their mothers had a history of jail time

Notes: ^a^RCT = Randomized Controlled Trial; ^b^HFA = Healthy Families America; NFP = Nurse Family Partnership; PAT = Parents as Teachers

None of the studies was specifically focused on CLS-involved families participating in FHV. Nevertheless, these studies revealed high rates of varying types of CLS involvement among FHV participants, their romantic partners/spouses, and in their family systems. For example, in their study examining parental risk factors and child abuse reports among a sample of parents participating in PAT, Duffy and colleagues (2015) reported that 19% of mothers and 59% of fathers had “criminal histories.” Similarly, Cullen and colleagues (2010) found that 64% of participants reported a family criminal history at baseline, although no additional information about what this entailed was included in either study. Some studies were more specific in their measurement of CLS. Black and colleagues (2006) found that 17% of mothers who had two children within 2 years reported a history of arrest and 11% had a history of being “jailed.”

CLS involvement among participants’ partners or spouses was also high. In their study examining adverse experiences in childhood and adulthood and mental health outcomes among participants in a non-MIECHV model program, Mersky and colleagues (2018) found that 48% of the sample reported the incarceration of a partner or spouse. Results from these quantitative studies indicate that CLS is a common adversity among families served by FHV. There is also some evidence that CLS-involved families may face additional challenges in the context of FHV services, including more family-related needs (e.g., Black et al. reported mothers with a second baby at baseline were more likely to report a history of arrest), and higher rates of mental health problems relative to participants without CLS involvement (e.g., Mersky et al., 2018; Traube et al., 2022).

Ten studies measured CLS involvement as an outcome variable (Table 3). These studies were published between 1997 (Olds et al., 1997) and 2022 (Petitclerc & Brooks-Gunn, 2022), with only two having been published in the last decade (Kitzman et al., 2019; Petitclerc & Brooks-Gunn, 2022). Among these ten studies, all but one focused on the outcomes of one generation (e.g., youth only or caregivers only); Shlafer and colleagues (2012) considered both maternal and youth outcomes simultaneously. Most of the studies focused on youths’ involvement in the juvenile justice system or CLS (e.g., youth arrest, delinquency), although a few considered caregivers’ CLS involvement over time (e.g., Grant et al., 2003; Olds et al., 1997, 2007). Five studies relied exclusively on self-report (e.g., youth reporting on their own behavior), but several studies included multiple sources of data, most often supplementing youth report with other sources (e.g., Eckenrode et al. (2001) collected youth report, parent report, and official records). The assessment of CLS contact varied among the studies, but most included multiple distinct measures (e.g., youths’ arrest, conviction, and delinquency). Most (seven of the ten studies) that examined CLS outcomes focused on participants in NFP. Longitudinal follow-up to assess CLS involvement ranged from 3 years (Grant et al., 2003) to 19 years (Eckenrode et al., 2001) after participating in FHV.

Within these studies, results indicated that FHV had some positive impacts on mothers’ and youths’ CLS outcomes. Among mothers, Grant et al. (2003) found a significant decrease in the number of mothers jailed from program exit to follow-up 3 years later. Olds et al. (1997) found that nurse-visited women had significantly fewer arrests 15 years later, but in a different trial, Olds et al. (2007) found no program effects on women’s arrests or being jailed after a 9-year follow-up. Among the studies that considered youths’ CLS outcomes, many demonstrated positive program impacts, but these impacts were moderated by different factors (e.g., sex, history of maltreatment). For example, Eckenrode et al. (2010) found FHV participation reduced the risk of youths’ rates of arrest and conviction, but only for girls. In contrast, Petitclerc and Brooks-Gunn (2022) found positive program effects for boys’ risk of being arrested, but not girls. Taken together, these studies demonstrate a small and nuanced evidence base for the impact of FHV on participants’ CLS outcomes.

## Discussion

This scoping review summarized the published research on the CLS experiences of FHV-enrolled families in the US. Among the 28 articles that met inclusion criteria, five were non-primary sources (i.e., systematic reviews or meta-analyses) and 23 were primary sources, only one of which was qualitative. The five review articles largely focused on papers describing well-known longitudinal studies of NFP (Olds et al., 1998, 1997, 2007), underscoring the lack of research on family CLS involvement in the broader home visiting literature and illuminating critical gaps that should be considered in future research. More specifically, our scoping review reveals a need for more contemporary research considering the CLS experiences of families participating in programs implementing models other than NFP.

Among the 22 quantitative articles that analyzed primary data, 12 were studies that measured family CLS involvement as a demographic variable. Across home visiting models, results indicate a high prevalence of parents and caregivers who have had contact with the CLS. These findings are perhaps unsurprising as most FHV programs aim to recruit families that are economically disadvantaged and at elevated risk of CLS involvement. Ten of the 22 quantitative studies examined CLS outcomes among FHV participants and only two of these studies were conducted in the last decade. This finding highlights an important gap in the existing literature and should encourage more contemporary research on this topic, particularly longitudinal research with repeated, short-term follow-up that seeks to enhance precision in estimating *precisely how* FHV might reduce family CLS or even disrupt the intergenerational transmission of CLS. Given the limitations of existing work, FHV research should prioritize examining pathways through which FHV might reduce the risk of CLS involvement for caregivers and their children over the life course, such as via enhanced parent–child attachment, improvements to emotional and behavior health, and enhanced connection to school and/or work for caregivers and children as they develop into adolescence and adulthood. Given the documented interconnections between early life health and socioeconomic disparities, crime, and CLS involvement across the life course, such research will benefit from multidisciplinary approaches that leverage complementary areas of expertise and bridge the gap between criminology and the health, social, and developmental sciences (Jackson & Vaughn, 2018).

There are a number of potential explanations for the dearth of evidence related to the CLS experiences of families receiving FHV services. First, most FHV programs were not designed specifically to serve families involved in the CLS, nor were they designed to be delinquency or crime prevention strategies. As such, much of the research to date has understandably focused on the primary intended outcomes of FHV (e.g., improved child well-being, reduced child abuse and neglect), rather than addressing CLS involvement. Further, although it is common for FHV programs to assess specific adversities (e.g., domestic violence, substance abuse), it is likely that many FHV programs do not screen for caregivers’ past or current involvement in the CLS. Recent national survey data suggests that fewer than half of home visitors are expected by their local programs to gather any data about family members’ involvement in the CLS (Jackson et al., 2024). This may be partially due to the fact MIECHV does not require programs to gather information about CLS; the closest performance indicator focuses on screening for domestic violence (Maternal & Child Health Bureau, 2024a, 2024b). In recent years, some FHV programs have begun collecting data on ACEs either for research or as part of broader initiatives to incorporate trauma-informed approaches to service delivery (Ballard et al., 2022; Cairone et al., 2017; Counts et al., 2017; Kemp et al., 2022; Mersky et al., 2017; Riggs et al., 2022). Thus, there may be opportunities to leverage existing data to better understand the prevalence of CLS involvement in FHV service population and ways that FHV programs may better serve these families.

Relatedly, identifying families who have experience with the CLS presents opportunities to partner to develop and test FHV intervention components that are most likely to be relevant and useful for impacted families. For example, families involved in the CLS often have court-ordered requirements (e.g., substance use testing, meetings with probation officers). They may also be interfacing with more than one court system (e.g., child protection cases in juvenile court; establishing parenting time in family court). Home visitors may be able to provide parents with support navigating these complex systems, including assistance with scheduling, transportation, and follow-up. Better understanding families’ experiences with the judicial system, at large, is an important area for future inquiry and could help inform practice recommendations.

The existing evidence does not tell us whether or how home visitors are modifying their work to reach, recruit, and serve CLS-involved families. This is a valuable area for future inquiry, particularly qualitative studies that might delve more deeply into home visitors’ experiences and practices. Given that CLS experiences are common among FHV families, it is important to better understand if existing models fit these families’ needs and, if not, how programs could be adapted. FHV data can also be linked with data from other child and family serving systems to facilitate observational and intervention research studies to better understand the impact of early life exposures on CLS involvement over the life course (Fantuzzo et al., 2017). Importantly, FHV will only benefit from this research if the findings are actionable and disseminated with clear practice and policy recommendations.

Across studies, there was a notable lack of precision in CLS language. In some instances, CLS terms were used interchangeably (e.g., “jail” and “incarceration”) and without specificity. For example, some studies assessed “CLS involvement;” this term could have varying interpretations to participants and researchers alike. Future research should include clear operational definitions of CLS involvement and—when possible—assess aspects of the CLS involvement known to impact family stability and child well-being, including the timing, during, and frequency of incarceration (Cho, 2010; Fox et al., 2023; Turney, 2022). There may also be untapped opportunities to establish common measures of CLS involvement and pool home visiting data from local programs or models to address questions of high priority to policy and practice. Relatedly, requiring MIECHV grantees to collect some standard measure of CLS involvement under the crime and violence performance indicator would significantly advance our understanding of this population and their needs. Given the important implications for families depending on the nature of involvement in the CLS (e.g., an arrest can be disruptive to the family system, but likely less so than a lengthy incarceration), these are important areas for future inquiry. Much could be learned from mixed methods approaches that consider families’ and home visitors’ experiences across different models.

The variation in families’ CLS experiences is likely to have implications for FHV service delivery. For example, families experiencing a father’s incarceration in prison versus a father with frequent experiences of arrest, release, and re-arrest are likely to have different needs and may benefit from the support of a home visitor in different ways (e.g., some felony convictions may result in an individual family member being barred from public housing). These collateral consequences of CLS involvement have important implications for home visitors, in that they may lead to increases in the number of families who have unstable housing and limited referral options. Improving precision in language and measurement of families’ CLS experiences and collaborating with home visiting staff and clients to identify, develop, and test components of FHV programs that align with the unique needs and preferences of CLS-involved families are valuable steps toward ensuring more effective programs (Jackson et al., 2024).

Our review also raises questions regarding the ways in which CLS involvement is conceptualized and operationalized in extant FHV research. Some options are to examine CLS involvement as an individual characteristic or as a community-level contextual factor, and each option has value. Yet, as noted by Davis and Kane (2016), a potential unintended consequence of heightening interest in individual-level ACEs, such as CLS involvement, is the risk of directing attention away from broader systems and social determinants of health and well-being. To that end, researchers and practitioners alike must be mindful of the need to consider multi-level determinants of CLS involvement and its implications for FHV access and outcomes. To date, limited FHV research has considered and included community-level indicators of violence or crime (Latimore et al., 2017; McGuigan et al., 2003). Future research could move beyond violence and crime indicators to consider, for example, how county-level arrest and incarceration rates are related to other social determinants of health (e.g., housing insecurity) that impact FHV program engagement.

Finally, our findings have implications for programs committed to serving families with diverse needs. The findings from our scoping review demonstrate that exposure to the CLS is a common experience among FHV families. Given our findings, all home visitors are likely to benefit from basic training related to the CLS (e.g., processes, key terminology) and its impacts on child and family health. Despite the potential benefits of training on the CLS and its impact on families, recent national survey data of home visitors suggest that only about one in three have received such a training, with roughly half of those receiving training reporting that it was required by their program (Jackson et al., 2024).

More research will also help inform whether FHV programs should consider training a cadre of home visitors to work with this population; home visitors with prior work experience with families impacted by the CLS may be able to provide unique insights and potentially partner with program supervisors to train and support such a cadre. We call for programs to consider the needs of these families by centering the voices of individuals directly impacted by incarceration in program development and delivery. This may include hiring home visitors who have their own lived experience with the CLS. Further, given the important role that community advisory councils play in FHV programming, our findings suggest that intentional partnerships between FHV programs and other local organizations serving CLS-impacted families (e.g., legal advocacy organizations) may be valuable.

### Limitations

This study is not without limitations. While scoping reviews help to summarize and quantify a body of literature, they are not designed to assess study quality. Further, unlike a meta-analysis, this scoping review does not draw any conclusions regarding the overall impacts of FHV on CLS outcomes. Additionally, the family policy and the CLS landscapes in the US are unique in many ways relative to other countries, and our exclusion criteria omitted studies from outside of the US, even though these studies could have valuable lessons for considering the intersections of FHV and the CLS. Our review is also limited by our decision to focus on published literature; it is possible that there are other relevant studies in gray literature that could inform this line of inquiry.

## Conclusions

Our review found relatively few studies that considered CLS involvement among FHV participants. Although limited, existing evidence does suggest that CLS involvement is a common experience among FHV participants. These findings have important implications for training home visitors to be prepared to sensitively and effectively work with CLS-involved families and highlight the need for additional research that tests whether targeted FHV strategies can reduce CLS involvement among participating families.

## Supplementary Information

Below is the link to the electronic supplementary material.

Supplementary file1 (DOCX 27 KB)
