# Supplementary material for: Criminal Legal System Experiences Among Families Receiving Home Visiting Services: A Scoping Review of the Literature
Source: Prev Sci. 2025 Mar 24;26(3):404–25. doi: 10.1007/s11121-025-01798-8 (PMC12064470; doi:10.1007/s11121-025-01798-8)
Supplement: Supplementary file 1 — Supplementary file1 (DOCX 27 KB) [file 11121_2025_1798_MOESM1_ESM.docx]

**Appendix 1.** Search Strategy.

### Databases:

1. Ovid Medline - 118
2. Embase - 205
3. PsycINFO - 101
4. CINAHL - 61
5. Social Services Abstracts - 71
6. Sociological Abstracts - 71
7. Criminal Justice Database - 108
8. Cochrane Library - 40

**Searches executed:** 3/15/2022

### Ovid Medline (118)

| **#** | **Search Statement** | **Results** |
| --- | --- | --- |
| 1 | exp Child/ or exp Infant/ or exp Family/ | 2,839,901 |
| 2 | (child* or infant* or newborn* or baby or babies or family or families or early childhood).ti,ab,kf. | 2,888,121 |
| 3 | 1 or 2 | 4,165,232 |
| 4 | exp House Calls/ or exp Home Care Services/ | 53,117 |
| 5 | (home adj2 visit*).ti,ab,kf. | 11,277 |
| 6 | 4 or 5 | 60,468 |
| 7 | 3 and 6 | 20,154 |
| 8 | ((evidence-based adj4 home visit*) or "Maternal, Infant, and Early Childhood Home Visiting" or MIECHV or "Attachment and Biobehavioral Catch-Up" or "Child First" or "Early Head Start Home-Based Option" or "Early Intervention Program for Adolescent Mothers" or "Family Check-Up For Children" or "Family Connects" or "Family Spirit" or "Health Access Nurturing Development Services" or "Healthy Beginnings" or "Healthy Families America" or "Home Instruction for Parents of Preschool Youngsters" or "Maternal Early Childhood Sustained Home-Visiting Program" or "Maternal Infant Health Program" or "Minding the Baby Home Visiting" or "Nurse-Family Partnership" or ("Play and Learning Strategies" adj2 Infant) or "SafeCare Augmented").ti,ab,kf. | 535 |
| 9 | 7 or 8 | 20,416 |
| 10 | exp Prisons/ or exp Jails/ or exp Prisoners/ or exp Criminal Law/ or exp Recidivism/ or exp Criminals/ or Juvenile Deliquency/ | 35,159 |
| 11 | (prison* or penitentiar* or jail* or gaol* or ((detention or correction*) adj2 (center* or facilit*)) or incarcerat* or imprison* or prisoner* or felon* or criminal* or convict* or offender* or arrest* or criminogen* or recidiv* or re-offend* or juvenile delinquen* or justice system*).ti,ab,kf. | 257,821 |
| 12 | 10 or 11 | 267,811 |
| 13 | 9 and 12 | 118 |

###

### Embase (205)

| **#** | **Search Statement** | **Results** |
| --- | --- | --- |
| 1 | exp Child/ or exp Infant/ or exp Family/ | 3,613,133 |
| 2 | (child* or infant* or newborn* or baby or babies or family or families or early childhood).ti,ab,kf. | 3,795,323 |
| 3 | 1 or 2 | 5,221,126 |
| 4 | exp home visit/ or Home Care/ | 71,891 |
| 5 | (home adj2 visit*).ti,ab,kf. | 15,243 |
| 6 | 4 or 5 | 81,519 |
| 7 | 3 and 6 | 25,373 |
| 8 | ((evidence-based adj4 home visit*) or "Maternal, Infant, and Early Childhood Home Visiting" or MIECHV or "Attachment and Biobehavioral Catch-Up" or "Child First" or "Early Head Start Home-Based Option" or "Early Intervention Program for Adolescent Mothers" or "Family Check-Up For Children" or "Family Connects" or "Family Spirit" or "Health Access Nurturing Development Services" or "Healthy Beginnings" or "Healthy Families America" or "Home Instruction for Parents of Preschool Youngsters" or "Maternal Early Childhood Sustained Home-Visiting Program" or "Maternal Infant Health Program" or "Minding the Baby Home Visiting" or "Nurse-Family Partnership" or ("Play and Learning Strategies" adj2 Infant) or "SafeCare Augmented").ti,ab,kf. | 635 |
| 9 | 7 or 8 | 25,743 |
| 10 | detention center/ or exp correctional facility/ or exp prisoner/ or exp Criminal Law/ or exp Criminal Justice/ or exp incarceration/ or exp Recidivism/ or exp offender/ or Juvenile Deliquency/ | 50,536 |
| 11 | (prison* or penitentiar* or jail* or gaol* or ((detention or correction*) adj2 (center* or facilit*)) or incarcerat* or imprison* or prisoner* or felon* or criminal* or convict* or offender* or arrest* or criminogen* or recidiv* or re-offend* or juvenile delinquen* or justice system*).ti,ab,kf. | 349,547 |
| 12 | 10 or 11 | 364,298 |
| 13 | 9 and 12 | 205 |

###

### PsycINFO (101)

| **#** | **Search Statement** | **Results** |
| --- | --- | --- |
| 1 | exp Family/ | 323,646 |
| 2 | (child* or infant* or newborn* or baby or babies or family or families or early childhood).ti,ab,id,hw. | 1,079,747 |
| 3 | 1 or 2 | 1,123,132 |
| 4 | exp Home Visiting Programs/ | 1,895 |
| 5 | (home adj2 visit*).ti,ab,id,hw. | 5,733 |
| 6 | 4 or 5 | 5,733 |
| 7 | 3 and 6 | 3,959 |
| 8 | ((evidence-based adj4 home visit*) or "Maternal, Infant, and Early Childhood Home Visiting" or MIECHV or "Attachment and Biobehavioral Catch-Up" or "Child First" or "Early Head Start Home-Based Option" or "Early Intervention Program for Adolescent Mothers" or "Family Check-Up For Children" or "Family Connects" or "Family Spirit" or "Health Access Nurturing Development Services" or "Healthy Beginnings" or "Healthy Families America" or "Home Instruction for Parents of Preschool Youngsters" or "Maternal Early Childhood Sustained Home-Visiting Program" or "Maternal Infant Health Program" or "Minding the Baby Home Visiting" or "Nurse-Family Partnership" or ("Play and Learning Strategies" adj2 Infant) or "SafeCare Augmented").ti,ab,id,hw. | 546 |
| 9 | 7 or 8 | 4,246 |
| 10 | exp Correctional Institutions/ or exp Incarceration/ or exp Prisoners/ or exp Criminal Justice/ or exp Criminal Law/ or exp Recidivism/ or exp Criminal Offenders/ or exp Juvenile Delinquency/ | 69,387 |
| 11 | (prison* or penitentiar* or jail* or gaol* or ((detention or correction*) adj2 (center* or facilit*)) or incarcerat* or imprison* or prisoner* or felon* or criminal* or convict* or offender* or arrest* or criminogen* or recidiv* or re-offend* or juvenile delinquen* or justice system*).ti,ab,id,hw. | 132,150 |
| 12 | 10 or 11 | 134,436 |
| 13 | 9 and 12 | 101 |

###

### CINAHL (61)

| **#** | **Search Statement** | **Results** |
| --- | --- | --- |
| S13 | S9 AND S12 | 61 |
| S12 | S10 OR S11 | 70,070 |
| S11 | TI ( (prison* or penitentiar* or jail* or gaol* or ((detention or correction*) N2 (center* or facilit*)) or incarcerat* or imprison* or prisoner* or felon* or criminal* or convict* or offender* or arrest* or criminogen* or recidiv* or re-offend* or juvenile delinquen* or justice system*) ) OR AB ( (prison* or penitentiar* or jail* or gaol* or ((detention or correction*) N2 (center* or facilit*)) or incarcerat* or imprison* or prisoner* or felon* or criminal* or convict* or offender* or arrest* or criminogen* or recidiv* or re-offend* or juvenile delinquen* or justice system*) ) | 63,336 |
| S10 | MH "Correctional Facilities" OR "Prisoners" OR "Criminal Justice" OR "Recidivism" OR "Public Offenders" OR "Juvenile Deliquency" OR "Juvenile Offenders" | 21,223 |
| S9 | S7 OR S8 | 5,570 |
| S8 | TI ( ((evidence-based N4 "home visit*") or "Maternal, Infant, and Early Childhood Home Visiting" or MIECHV or "Attachment and Biobehavioral Catch-Up" or "Child First" or "Early Head Start Home-Based Option" or "Early Intervention Program for Adolescent Mothers" or "Family Check-Up For Children" or "Family Connects" or "Family Spirit" or "Health Access Nurturing Development Services" or "Healthy Beginnings" or "Healthy Families America" or "Home Instruction for Parents of Preschool Youngsters" or "Maternal Early Childhood Sustained Home-Visiting Program" or "Maternal Infant Health Program" or "Minding the Baby Home Visiting" or "Nurse-Family Partnership" or ("Play and Learning Strategies" N2 Infant) or "SafeCare Augmented") ) OR AB ( ((evidence-based N4 "home visit*") or "Maternal, Infant, and Early Childhood Home Visiting" or MIECHV or "Attachment and Biobehavioral Catch-Up" or "Child First" or "Early Head Start Home-Based Option" or "Early Intervention Program for Adolescent Mothers" or "Family Check-Up For Children" or "Family Connects" or "Family Spirit" or "Health Access Nurturing Development Services" or "Healthy Beginnings" or "Healthy Families America" or "Home Instruction for Parents of Preschool Youngsters" or "Maternal Early Childhood Sustained Home-Visiting Program" or "Maternal Infant Health Program" or "Minding the Baby Home Visiting" or "Nurse-Family Partnership" or ("Play and Learning Strategies" N2 Infant) or "SafeCare Augmented") ) | 405 |
| S7 | (S4 OR S5) AND (S3 AND S6) | 5,390 |
| S6 | S4 OR S5 | 11,734 |
| S5 | TI home N2 visit* OR AB home N2 visit* | 8,120 |
| S4 | MH Home Visits | 6,598 |
| S3 | S1 OR S2 | 1,095,539 |
| S2 | TI ( (child* or infant* or newborn* or baby or babies or family or families or "early childhood") ) OR AB ( (child* or infant* or newborn* or baby or babies or family or families or "early childhood") ) | 853,569 |
| S1 | MH child or infant or family | 632,087 |

###

### Social Services Abstracts (71)

| **#** | **Search Statement** | **Results** |
| --- | --- | --- |
| 1 | ((ti((child* OR infant* OR newborn* OR baby OR babies OR family OR families OR "early childhood")) OR ab((child* OR infant* OR newborn* OR baby OR babies OR family OR families OR "early childhood")) OR su((child* OR infant* OR newborn* OR baby OR babies OR family OR families OR "early childhood"))) AND ((ti((home NEAR/2 visit*)) OR ab((home NEAR/2 visit*)) OR su((home NEAR/2 visit*))) OR (ti(((evidence-based NEAR/4 "home visit*") OR "Maternal, Infant, and Early Childhood Home Visiting" OR MIECHV OR "Attachment and Biobehavioral Catch-Up" OR "Child First" OR "Early Head Start Home-Based Option" OR "Early Intervention Program for Adolescent Mothers" OR "Family Check-Up For Children" OR "Family Connects" OR "Family Spirit" OR "Health Access Nurturing Development Services" OR "Healthy Beginnings" OR "Healthy Families America" OR "Home Instruction for Parents of Preschool Youngsters" OR "Maternal Early Childhood Sustained Home-Visiting Program" OR "Maternal Infant Health Program" OR "Minding the Baby Home Visiting" OR "Nurse-Family Partnership" OR ("Play and Learning Strategies" NEAR/2 Infant) OR "SafeCare Augmented")) OR ab(((evidence-based NEAR/4 "home visit*") OR "Maternal, Infant, and Early Childhood Home Visiting" OR MIECHV OR "Attachment and Biobehavioral Catch-Up" OR "Child First" OR "Early Head Start Home-Based Option" OR "Early Intervention Program for Adolescent Mothers" OR "Family Check-Up For Children" OR "Family Connects" OR "Family Spirit" OR "Health Access Nurturing Development Services" OR "Healthy Beginnings" OR "Healthy Families America" OR "Home Instruction for Parents of Preschool Youngsters" OR "Maternal Early Childhood Sustained Home-Visiting Program" OR "Maternal Infant Health Program" OR "Minding the Baby Home Visiting" OR "Nurse-Family Partnership" OR ("Play and Learning Strategies" NEAR/2 Infant) OR "SafeCare Augmented"))))) AND (prison* or penitentiar* or jail* or gaol* or ((detention or correction*) NEAR/2 (center* or facilit*)) or incarcerat* or imprison* or prisoner* or felon* or criminal* or convict* or offender* or arrest* or criminogen* or recidiv* or re-offend* or "juvenile delinquen*" or "justice system*") | 71 |

### Sociological Abstracts (71)

| **#** | **Search Statement** | **Results** |
| --- | --- | --- |
| 1 | ((ti((child* OR infant* OR newborn* OR baby OR babies OR family OR families OR "early childhood")) OR ab((child* OR infant* OR newborn* OR baby OR babies OR family OR families OR "early childhood")) OR su((child* OR infant* OR newborn* OR baby OR babies OR family OR families OR "early childhood"))) AND ((ti((home NEAR/2 visit*)) OR ab((home NEAR/2 visit*)) OR su((home NEAR/2 visit*))) OR (ti(((evidence-based NEAR/4 "home visit*") OR "Maternal, Infant, and Early Childhood Home Visiting" OR MIECHV OR "Attachment and Biobehavioral Catch-Up" OR "Child First" OR "Early Head Start Home-Based Option" OR "Early Intervention Program for Adolescent Mothers" OR "Family Check-Up For Children" OR "Family Connects" OR "Family Spirit" OR "Health Access Nurturing Development Services" OR "Healthy Beginnings" OR "Healthy Families America" OR "Home Instruction for Parents of Preschool Youngsters" OR "Maternal Early Childhood Sustained Home-Visiting Program" OR "Maternal Infant Health Program" OR "Minding the Baby Home Visiting" OR "Nurse-Family Partnership" OR ("Play and Learning Strategies" NEAR/2 Infant) OR "SafeCare Augmented")) OR ab(((evidence-based NEAR/4 "home visit*") OR "Maternal, Infant, and Early Childhood Home Visiting" OR MIECHV OR "Attachment and Biobehavioral Catch-Up" OR "Child First" OR "Early Head Start Home-Based Option" OR "Early Intervention Program for Adolescent Mothers" OR "Family Check-Up For Children" OR "Family Connects" OR "Family Spirit" OR "Health Access Nurturing Development Services" OR "Healthy Beginnings" OR "Healthy Families America" OR "Home Instruction for Parents of Preschool Youngsters" OR "Maternal Early Childhood Sustained Home-Visiting Program" OR "Maternal Infant Health Program" OR "Minding the Baby Home Visiting" OR "Nurse-Family Partnership" OR ("Play and Learning Strategies" NEAR/2 Infant) OR "SafeCare Augmented"))))) AND (prison* or penitentiar* or jail* or gaol* or ((detention or correction*) NEAR/2 (center* or facilit*)) or incarcerat* or imprison* or prisoner* or felon* or criminal* or convict* or offender* or arrest* or criminogen* or recidiv* or re-offend* or "juvenile delinquen*" or "justice system*") | 71 |

###

### Criminal Justice Database (108)

| **#** | **Search Statement** | **Results** |
| --- | --- | --- |
| 1 | ((ti((child* OR infant* OR newborn* OR baby OR babies OR family OR families OR "early childhood")) OR ab((child* OR infant* OR newborn* OR baby OR babies OR family OR families OR "early childhood")) OR su((child* OR infant* OR newborn* OR baby OR babies OR family OR families OR "early childhood"))) AND ((ti((home NEAR/2 visit*)) OR ab((home NEAR/2 visit*)) OR su((home NEAR/2 visit*))) OR (ti(((evidence-based NEAR/4 "home visit*") OR "Maternal, Infant, and Early Childhood Home Visiting" OR MIECHV OR "Attachment and Biobehavioral Catch-Up" OR "Child First" OR "Early Head Start Home-Based Option" OR "Early Intervention Program for Adolescent Mothers" OR "Family Check-Up For Children" OR "Family Connects" OR "Family Spirit" OR "Health Access Nurturing Development Services" OR "Healthy Beginnings" OR "Healthy Families America" OR "Home Instruction for Parents of Preschool Youngsters" OR "Maternal Early Childhood Sustained Home-Visiting Program" OR "Maternal Infant Health Program" OR "Minding the Baby Home Visiting" OR "Nurse-Family Partnership" OR ("Play and Learning Strategies" NEAR/2 Infant) OR "SafeCare Augmented")) OR ab(((evidence-based NEAR/4 "home visit*") OR "Maternal, Infant, and Early Childhood Home Visiting" OR MIECHV OR "Attachment and Biobehavioral Catch-Up" OR "Child First" OR "Early Head Start Home-Based Option" OR "Early Intervention Program for Adolescent Mothers" OR "Family Check-Up For Children" OR "Family Connects" OR "Family Spirit" OR "Health Access Nurturing Development Services" OR "Healthy Beginnings" OR "Healthy Families America" OR "Home Instruction for Parents of Preschool Youngsters" OR "Maternal Early Childhood Sustained Home-Visiting Program" OR "Maternal Infant Health Program" OR "Minding the Baby Home Visiting" OR "Nurse-Family Partnership" OR ("Play and Learning Strategies" NEAR/2 Infant) OR "SafeCare Augmented"))))) AND (prison* or penitentiar* or jail* or gaol* or ((detention or correction*) NEAR/2 (center* or facilit*)) or incarcerat* or imprison* or prisoner* or felon* or criminal* or convict* or offender* or arrest* or criminogen* or recidiv* or re-offend* or "juvenile delinquen*" or "justice system*") | 108 |

### Cochrane Library (40)

| **ID** | **Search** | **Hits** |
| --- | --- | --- |
| #1 | MeSH descriptor: [Child] explode all trees | 60484 |
| #2 | MeSH descriptor: [Infant] explode all trees | 34437 |
| #3 | MeSH descriptor: [Family] explode all trees | 10385 |
| #4 | (child* or infant* or newborn* or baby or babies or family or families or "early childhood") | 258882 |
| #5 | #1 or #2 or #3 or #4 | 259757 |
| #6 | MeSH descriptor: [House Calls] explode all trees | 578 |
| #7 | MeSH descriptor: [Home Care Services] explode all trees | 2561 |
| #8 | ((evidence-based NEAR/4 "home visit*") or "Maternal, Infant, and Early Childhood Home Visiting" or MIECHV or "Attachment and Biobehavioral Catch-Up" or "Child First" or "Early Head Start Home-Based Option" or "Early Intervention Program for Adolescent Mothers" or "Family Check-Up For Children" or "Family Connects" or "Family Spirit" or "Health Access Nurturing Development Services" or "Healthy Beginnings" or "Healthy Families America" or "Home Instruction for Parents of Preschool Youngsters" or "Maternal Early Childhood Sustained Home-Visiting Program" or "Maternal Infant Health Program" or "Minding the Baby Home Visiting" or "Nurse-Family Partnership" or ("Play and Learning Strategies" NEAR/2 Infant) or "SafeCare Augmented") | 357 |
| #9 | #6 or #7 or #8 | 3384 |
| #10 | #5 and #9 | 1440 |
| #11 | MeSH descriptor: [Prisoners] explode all trees | 344 |
| #12 | MeSH descriptor: [Prisons] explode all trees | 138 |
| #13 | MeSH descriptor: [Jails] explode all trees | 4 |
| #14 | MeSH descriptor: [Criminal Law] explode all trees | 97 |
| #15 | MeSH descriptor: [Recidivism] explode all trees | 25 |
| #16 | MeSH descriptor: [Criminals] explode all trees | 121 |
| #17 | MeSH descriptor: [Juvenile Delinquency] explode all trees | 247 |
| #18 | (prison* or penitentiar* or jail* or gaol* or ((detention or correction*) NEAR/2 (center* or facilit*)) or incarcerat* or imprison* or prisoner* or felon* or criminal* or convict* or offender* or arrest* or criminogen* or recidiv* or re-offend* or "juvenile delinquen*" or "justice system*") | 13798 |
| #19 | #11 or #12 or #13 or #14 or #15 or #16 or #17 or #18 | 13944 |
| #20 | #10 and #19 | 40 |
